# FMCW Radar Estimation Algorithm with High Resolution and Low Complexity Based on Reduced Search Area

**DOI:** 10.3390/s22031202

**Published:** 2022-02-05

**Authors:** Bong-Seok Kim, Youngseok Jin, Jonghun Lee, Sangdong Kim

**Affiliations:** 1 Division of Automotive Technology, Daegu Gyeongbuk Institute of Science and Technology (DGIST), Daegu 42988, Korea; remnant@dgist.ac.kr (B.-S.K.); ysjin@dgist.ac.kr (Y.J.); jhlee@dgist.ac.kr (J.L.); 2 Department of Interdisciplinary Engineering, Daegu Gyeongbuk Institute of Science and Technology (DGIST), Daegu 42988, Korea

**Keywords:** FMCW radar, estimation, super resolution, low complexity, search area

## Abstract

We propose a frequency-modulated continuous wave (FMCW) radar estimation algorithm with high resolution and low complexity. The fast Fourier transform (FFT)-based algorithms and multiple signal classification (MUSIC) algorithms are used as algorithms for estimating target parameters in the FMCW radar systems. FFT-based and MUSIC algorithms have tradeoff characteristics between resolution performance and complexity. While FFT-based algorithms have the advantage of very low complexity, they have the disadvantage of a low-resolution performance; that is, estimating multiple targets with similar parameters as a single target. On the other hand, subspace-based algorithms have the advantage of a high-resolution performance, but have a problem of very high complexity. In this paper, we propose an algorithm with reduced complexity, while achieving the high-resolution performance of the subspace-based algorithm by utilizing the advantages of the two algorithms; namely, the low-complexity advantage of FFT-based algorithms and the high-resolution performance of the MUSIC algorithms. The proposed algorithm first reduces the amount of data used as input to the subspace-based algorithm by using the estimation results obtained by FFT. Secondly, it significantly reduces the range of search regions considered for pseudo-spectrum calculations in the subspace-based algorithm. The simulation and experiment results show that the proposed algorithm achieves a similar performance compared with the conventional and low complexity MUSIC algorithms, despite its considerably lower complexity.

## 1. Introduction

Recently, there has been growing interest in radar sensors in various fields, such as vehicles, surveillance, defense, etc. This is because radar sensors are robust under several conditions such as humidity, strong light, and bad weather [1,2,3,4,5,6]. In particular, among several kinds of radar sensors, frequency-modulated continuous wave (FMCW) radar systems are widely employed due to their low costs and low power consumption, even with a small size [7,8,9,10,11,12,13,14,15,16]. The FMCW radar have many merits compared with the ultra-wide band pulse radar, such as the low transmitted power and performance to simultaneously estimate the range and velocity of targets. In addition, due to the significantly lowered frequency band after mixing, the circuit complexity of the hardware is simplified [17,18].

In the FMCW radar systems, fast Fourier transform (FFT) is employed as a representative technique for estimating the distance, the velocity, and the angle of targets [9,10,11,12,13,14]. This is because the frequency of the sine wave, the so-called beat-frequency, is used for distance estimation in the FMCW radar systems. As is well known, FFT-based algorithms provide the same output as discrete Fourier transform, and have significantly lower complexity. For instance, in [14], FFT was employed in order to estimate the range of targets for surveillance applications. In [14], in order to solve the blind speed problem, an FFT was performed on the difference between two ramp signals randomly selected. In [15], in order to reduce the complexity while improving the angular resolution, the FFT was utilized after extrapolation. For extrapolation, the authors used the multiplications among multiple received signals. In [10,11,12,13], multidimensional, i.e., 2D and 3D FFT-based algorithms, were used for the estimation of target information. To further reduce computational complexity, in [11], they reversed the order of the FFT for distance estimation and FFT for velocity estimation. In [16], in order to further reduce the redundant complexity, they selected one method between the FFT and the partial discrete Fourier transform (DFT) for velocity estimation, based on the number estimated targets by FFT for range estimation. However, due to the limitations on available bandwidth, FFT-based algorithms might not achieve the fine resolution of estimation results. This means that, in a case where the distances or velocities of the multiple targets are similar, it might be estimated as if they were a single target, despite being multiple targets.

In FMCW radar systems, to solve this problem of estimators based on FFT, subspace-based algorithms have been studied [19,20,21,22,23,24,25,26,27,28,29,30,31,32]. In [19,20], Estimation of Signal Parameters via Rotational Invariance Techniques (ESPRIT)-based algorithms are employed to accurately estimate the direction of arrival (DOA) of targets. In [19], they estimated the DOA of targets by employing the ESPRIT algorithm. In particular, they constructed a covariance matrix into a Toeplitz matrix and symmetrical structure. Thus, they achieved higher angle resolution compared to the conventional ESPRIT algorithm. In [20], the authors tried to improve the angle resolution by employing the ESPRIT algorithm in FMCW radar systems. They proposed an ESPRIT processor with a scalable number of antennas, and designed the FPGA-based systems to verify the performance.

In [23,24,25,26,27,28,29,30,31,32,33,34,35,36], multiple signal classification (MUSIC) algorithms, which are representative subspace-based algorithms, have been proposed. In [25], authors have proposed the distributed 2D MUSIC algorithm with coordinated transformation in a distributed way. In this paper, each radar performed 2D MUSIC with its own received signal in the transformed coordinates. In [26,27], they have extended the antenna array structure from 1D to 2D to perform the joint 3D estimation of range, azimuth, and elevation angles. In [26], for 3D MUSIC spectrum calculation, they employed the augmented 2D steering vector and connected two 2D steering vectors in a specific way. In [27], they have proposed the 3D MUSIC algorithm with auto-pairing by employing 3D shift-invariant stacked Hankel matrix, which consists of 1D Hankel matrices. In [29,32,34], authors employed the FFT-estimation to reduce the computational complexity of MUSIC algorithm for FMCW radar systems. In [34], first, range estimation based on FFT is performed, and thus range bins are obtained. Then, a 1D MUSIC algorithm is performed to estimate Doppler frequency with high resolution; only for the obtained range bins where the targets exist. By doing so, computational complexity is reduced compared to the 2D MUSIC algorithm. However, it is possible to estimate a plurality of adjacent targets as a single target, which may degrade the performance of 1D MUSIC performed for Doppler estimation, because the range estimation is based on FFT with low resolution. In [29,32], authors tried to reduce the redundant complexity of the MUSIC algorithm by decreasing the number of input samples based on FFT estimation results. The conditions in which the resolution performance of the range and DOA is not degraded are presented, and thus, the overall complexity is reduced by decreasing the number of inputs of the MUSIC algorithm based on the FFT estimation. However, there are still drawbacks. In the step of pseudo-spectrum calculations to scan in MUSIC algorithms, they considered all the regions regardless of the information of the targets. Hence, there were a lot of unnecessary operations included, because the targets are mostly limited to specific regions. If the number of types of parameters considered increases, that is, if the number of dimensions increases, the resulting complexity increase becomes more critical.

In this paper, the proposed algorithm further reduces the redundant complexity by reducing the number of redundant samples in the input step of the MUSIC algorithm, and by limiting the region considered in the process of the pseudo spectrum calculation, where targets are located. In order to search the location of targets, we utilized the results of the FFT-based estimation with low complexity. Moreover, by considering two kinds of parameters, i.e., the range and DOA, 2D data are considered in this paper. By complexity analysis, we illustrated how much computation was saved by the proposed algorithm for various parameters. Moreover, the simulation and experiment results show that the proposed algorithm achieves a similar performance compared to, not only the conventional MUSIC algorithm, but also the low complexity MUSIC algorithm [29], despite its considerably lower complexity. Furthermore, we derive the optimal number of samples in the antenna domain according to the FFT-estimation result. Thus, the expression that is complicated in [32] is expressed as a simple form.

The remainder of this paper is organized as follows. Section 2 describes the system model of the FMCW radar system and data structure. In Section 3, 2D FFT and 2D MUSIC algorithms are addressed. Then, in Section 4, the proposed algorithms are illustrated. In Section 5, the performance and complexity of the proposed algorithm are analyzed and verified through simulations and experiments using 24 GHz FMCW radar systems. Finally, Section 6 concludes this paper and Section 7 deals with discussion and further studies.

## 2. System Model and Data Structure

**Figure 1:**
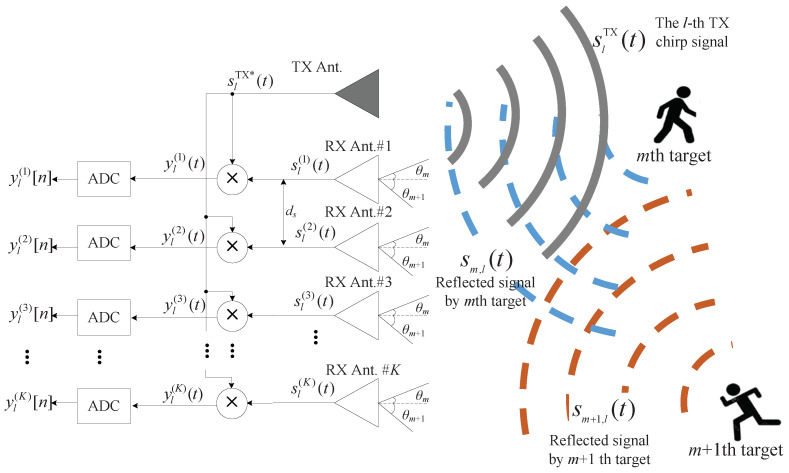
System model of the considered FMCW radar (1 TX and *K* RX antennas).

In this section, we address the system model and data structure considered in this paper. We consider the FMCW radar system, which has one transmitted (TX) antenna and *K* received (RX) antennas, as shown in Figure 1.

**Figure 2:**
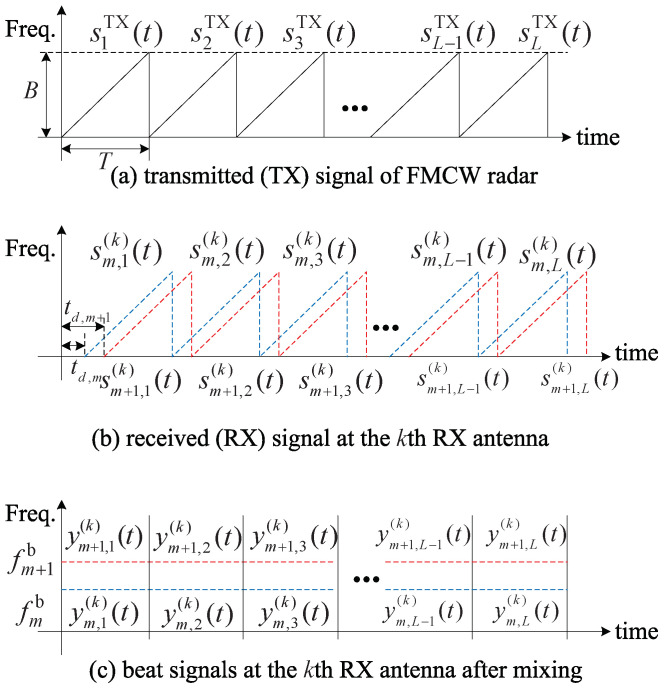
Structure of TX, RX, and the beat signals of FMCW radar.

The TX FMCW radar signal $s^{\mathrm{TX}}\left(t\right)$ is radiated from TX antenna, i.e., $s^{\mathrm{TX}}\left(t\right)$ is represented by:

(1) $$\begin{array}{c} s^{\mathrm{TX}}\left(t\right)=\exp\left(j2\pi \left(f_{c}t+\int_{0}^{t}\mu \tau \;d\tau \right)\right),\;\;\mathrm{for}\;\;\;0\leq t\leq T \end{array}$$

where $f_{c}$ is the central frequency of FMCW radar system, μ is a slope that linearly increases with a slope according to time τ during one sweep duration *T*, i.e., μ=B/T where *B* is the analog bandwidth of FMCW radar system as shown in Figure 2a. The TX signal $s^{\mathrm{TX}}\left(t\right)$ is total *L* times transmitted and thus the *l*th TX signal is denoted by $s_{l}^{\mathrm{TX}}\left(t\right)=s^{\mathrm{TX}}\left(t-\left(l-1\right)T\right)\;\;\mathrm{for}\;\;1\leq l\leq L$. We consider that the TX signal is reflected by *M* targets and is received *K* RX antennas as shown in Figure 2b. In Figure 1 and Figure 2, for simplicity, the two targets are considered. The reflected signal by the *m*th target $s_{m,l}\left(t\right)$ is expressed as follows:

(2) $$\begin{array}{c} s_{m,l}\left(t\right)={\tilde{\alpha }}_{m}s_{l}^{\mathrm{TX}}\left(t-t_{d,m}\right)\exp\left(j2\pi f_{D,m}\left(\left(l-1\right)T\right)\right) \end{array}$$

where ${\tilde{\alpha }}_{m}$ is the complex amplitude of the reflected signal by the *m*th target, $t_{d,m}$ is the delay time due to the range between radar and the *m*th target, and $f_{D,m}$ is the Doppler frequency, due to the moving velocity of the *m*th target. The reflected signal $s_{m,l}\left(t\right)$ is received at the *k*th RX antenna, and the RX signal $s_{l}^{\left(k\right)}\left(t\right)$ is expressed as:

(3) $$\begin{array}{c} s_{l}^{\left(k\right)}\left(t\right)=\sum_{m=1}^{M}{\tilde{\alpha }}_{m}s_{m,l}\left(t\right)\exp\left(\frac{j2\pi d_{s}\left(k-1\right)\sin\theta_{m}}{\lambda }\right)+{\tilde{z}}_{l}^{\left(k\right)}\left(t\right) \end{array}$$

where $d_{s}$ is the distance (space) between adjacent RX antennas, $\theta_{m}$ is DOA of the *m*th target as in Figure 1, λ is the wavelength, and ${\tilde{z}}_{l}^{\left(k\right)}\left(t\right)$ is the additive white Gaussian noise (AWGN) component at the *k*th RX antenna. As shown in the left side in Figure 1, the RX signals are mixed by the conjugate of TX signal, and thus the mixed signal, so-called ‘beat signal’, is denoted by $y_{l}^{\left(k\right)}\left(t\right)$, i.e., $y_{l}^{\left(k\right)}\left(t\right)=s_{l}^{\left(k\right)}\left(t\right)s^{*\mathrm{TX}}\left(t\right)$ for 1≤k≤K and is expressed as:

(4) $$\begin{array}{ccc} y_{l}^{\left(k\right)}\left(t\right) & = & s_{l}^{\left(k\right)}\left(t\right)\times s^{*\mathrm{TX}}\left(t\right)\\ & = & \sum_{m=1}^{M}\underset{\;≜{\dot{\alpha }}_{m,l}}{\underbrace{{\tilde{\alpha }}_{m,l}\exp(-j(2\pi f_{c}t_{d,m}-\mu t_{d,m}^{2}/2))}}\exp\left(-j2\pi \mu t_{d,m}t\right)\\ & \times & \exp\left(j2\pi f_{D,m}\left(\left(l-1\right)T\right)\right)\exp\left(\frac{j2\pi d\left(k-1\right)\sin\theta_{m}}{\lambda }\right)+\underset{\;≜{z_{l}^{\left(k\right)}}_{\left(t\right)}}{\underbrace{{\tilde{z}}_{l}^{\left(k\right)}\left(t\right)\times S_{l}^{*\mathrm{TX}}\left(t\right)}}. \end{array}$$

Assuming $d_{s}=\lambda /2$, (4) is simply rewritten in terms of range, Doppler, DOA, and noise as follows:

(5) $$\begin{array}{c} \begin{array}{ccc} y_{l}^{\left(k\right)}\left(t\right) & = & \sum_{m=1}^{M}{\dot{\alpha }}_{m,l}\underset{\;\mathrm{range}}{\underbrace{\exp\left(-j2\pi \mu t_{d,m}t\right)}}\underset{\mathrm{Doppler}}{\underbrace{\exp\left(j2\pi f_{D,m}\left(\left(l-1\right)T\right)\right)}}\\ & \times & \underset{\mathrm{DOA}}{\underbrace{\exp\left(j\pi \left(k-1\right)\sin\theta_{m}\right)}}+\underset{\mathrm{AWGN}}{\underbrace{z_{l}^{\left(k\right)}\left(t\right)}}. \end{array} \end{array}$$

As shown in Figure 1, the analog beat signal $y_{l}^{\left(k\right)}\left(t\right)$ is converted to the digital signal. The analog to digital convert (ADC) beat signal $y_{l}^{\left(k\right)}\left[n\right]$ is expressed as follows:

(6) $$\begin{array}{ccc} y_{l}^{\left(k\right)}\left[n\right] & = & \sum_{m=1}^{M}{\dot{\alpha }}_{m,l}\underset{≜x_{m}\left[n\right]}{\underbrace{\exp\left(-j2\pi \mu t_{d,m}nt_{s}\right)}}\underset{≜v_{m}^{\left(l\right)}}{\underbrace{\exp\left(j2\pi f_{D,m}\left(\left(l-1\right)T\right)\right)}}\\ & \times & \underset{≜\psi_{m}^{k}}{\underbrace{\exp\left(j\pi \left(k-1\right)\sin\theta_{m}\right)}}+\underset{≜z_{l}^{\left(k\right)}\left[n\right]}{\underbrace{z^{\left(k\right)}\left[nt_{s}\right]}}\;\;\;\;\mathrm{for}\;1\leq n\leq N_{s} \end{array}$$

where $t_{s}\left(=1/f_{s}\right)$ is sampling time interval, $f_{s}$ is the sampling frequency, and $N_{s}$ is the number of total samples, i.e., $\left\lfloor N_{s}=T/t_{s}\right\rfloor$ where ⌊·⌋ is the floor operator to the nearest integer number. Figure 2c shows an example of the two beat signals at the *k*th RX antenna after mixing. The *m*th beat frequency fmb increases as the delay increases, and, thus, the *m*th beat frequency that arrives earlier is lower compared with the *m* + 1th beat frequency that arrives later. In the FMCW radar system, the delay time is estimated by estimating this beat frequency, and the range of the *m*th target ${\hat{d}}_{m}$ is estimated based on the estimated delay time as follows:

(7) $$\begin{array}{c} {\hat{d}}_{m}=\frac{t_{d,m}\times c}{2}=\frac{f_{b,m}\times c}{2\mu }. \end{array}$$

**Figure 3:**
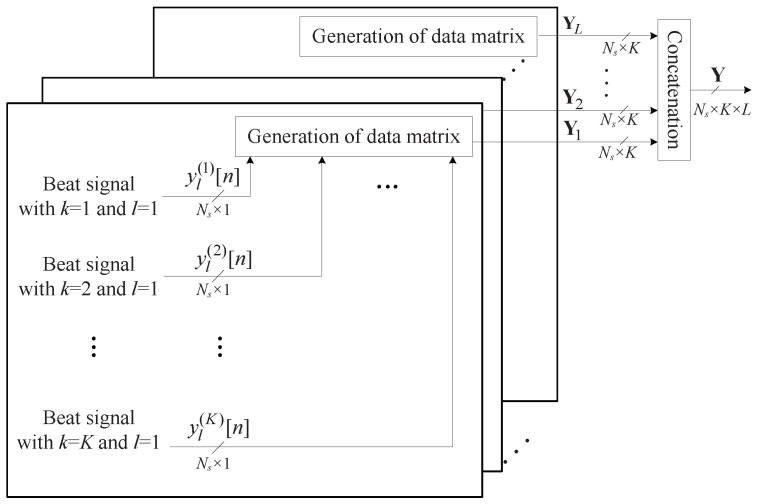
Generation process data matrix based on time, antenna, and chirp domains.

**Figure 4:**
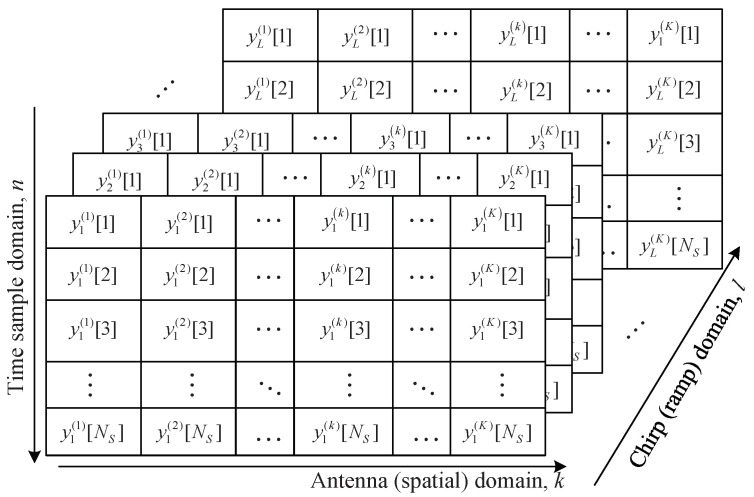
Structure of data matrix with respect to time, antenna, and chirp domains.

Figure 3 illustrates the process structure to obtain the 3D data matrices with respect to time, antenna, and the chirp domains. The total *K* beat signals of length $N_{s}$ are concatenated to form a matrix of $N_{s}\times K$. Let us denote $Y_{l}$ by the 2D data matrix at the *l*th chirp. Then, the 3D data matrix is finally generated by concatenating $Y_{l}$ for 1≤l≤L. Figure 4 shows the structure of the 3D data matrix obtained. The range, angle, and velocity are estimated through frequency estimations of these data matrices in the time domain, antenna domain, and chirp domain, respectively [16]. If the estimators based on 3D FFT are used, while range, speed, and DOA can be estimated with low complexity, there is a problem, in that adjacent parameters cannot be distinguished, due to the characteristics of the low resolution of FFT. On the other hand, by employing the subspace-based algorithms instead of FFT, the problem of the degradation of resolution can be solved, but the computational complexity significantly increases. In other words, there is a tradeoff between resolution and complexity between the two kinds of algorithms. In the next section, we address these algorithms, and then we propose a suboptimal solution to the problems of two kinds of algorithms. However, for convenience and efficient explanation, this paper focuses only on the estimation of range and DOA.

## 3. 2D FFT and 2D MUSIC Algorithms for FMCW Radar

### 3.1. 2D FFT Algorithm

This section describes the 2D FFT algorithm for FMCW radar. The FFT algorithm is the most widely used frequency estimation algorithm. The FFT significantly reduces complexity while providing the same output as the DFT by avoiding the redundant computation complexity in the DFT operation. Therefore, the FFT algorithm is considered one of the representative estimation algorithms in the FMCW radar systems. First, for range estimation, the $N_{R}\times K$ range bins are obtained by performing $N_{R}$ point FFT operation for 1≤k≤K where $N_{R}$ is the size of FFT for range estimation. The *p*th FFT output on $y_{l}^{k}\left[n\right]$, i.e., the *u*th range bins, is denoted by $Y_{l}^{\left(k\right)}\left[p\right]$, and it is calculated as follows:

(8) $$\begin{array}{c} Y_{l}^{\left(k\right)}\left[p\right]=\left\{\begin{array}{c} \sum_{n=1}^{N_{s}}y_{l}^{\left(k\right)}\left[n\right]\exp\left(\frac{-j2\pi (n-1)(p-1)}{N_{R}}\right),\;\mathrm{for}\;\;\;1\leq p\leq N_{s}\\ 0,\;\;\;\;\mathrm{for}\;\;\;N_{s}+1\leq p\leq N_{R}. \end{array}\right. \end{array}$$

Secondly, for DOA estimation, $N_{A}$ point FFT operation on $N_{R}\times K$ range bins is performed in the antenna domain. That is, the *q*th FFT output on $Y_{l}^{\left(k\right)}\left[p\right]$ is denoted by $Y_{l}^{\left(q\right)}\left[p\right]$, and it is calculated as follows:

(9) $$\begin{array}{c} Y_{l}^{\left(q\right)}\left[u\right]=\left\{\begin{array}{c} \sum_{k=1}^{K}Y_{l}^{\left(k\right)}\left[p\right]\exp\left(\frac{-j2\pi (k-1)(q-1)}{N_{A}}\right),\;\mathrm{for}\;\;\;1\leq q\leq K\\ 0,\;\;\;\;\mathrm{for}\;\;\;K+1\leq q\leq N_{A}. \end{array}\right. \end{array}$$

Then, the peak detection on the magnitude of $Y_{l}^{\left(q\right)}\left[p\right]$, i.e., $|Y_{l}^{\left(q\right)}\left[p\right]|$ is performed, and thus the *M* peak pairs are obtained, i.e., $\left(p_{m},q_{m}\right)$ for 1≤m≤M. From these peak pairs, the range and DOA are estimated.

**Figure 5:**
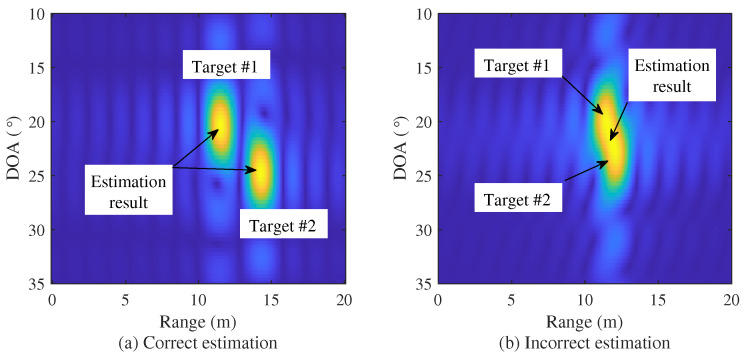
Example of correct and incorrect cases of FFT estimation.

As shown in Figure 5, however, it might be incorrectly estimated as a single target, even though there are multiple adjacent targets, since the resolution of the FFT operation is low. The circles in Figure 5 are the actual range and DOA of targets. In this case, super-resolution algorithms with a higher resolution than the FFT are required. In Section 3.2, the 2D MUSIC algorithm is introduced as a representative algorithm of super-resolution algorithms.

### 3.2. 2D MUSIC Algorithm

**Figure 6:**
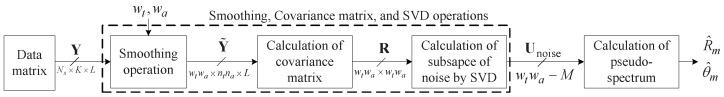
Structure of the conventional 2D MUSIC algorithm with respect to 3D data matrix.

**Figure 7:**
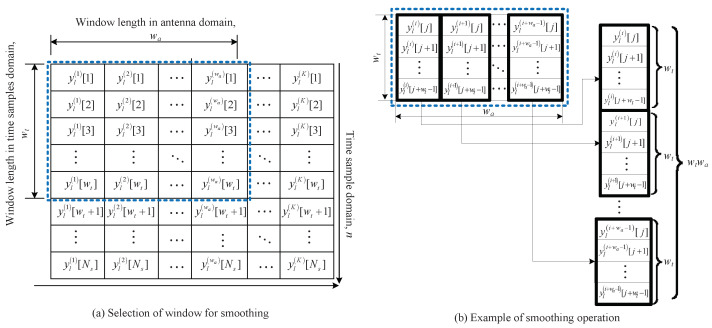
Example of smoothing algorithm to increase rank of correlation matrix for 2D MUSIC algorithm.

This section describes the 2D MUSIC algorithms for the FMCW radar. Figure 6 illustrates the structure of the 2D MUSIC algorithm. First, the smoothing operation is performed on the data matrix Y, in order to increase the rank of the matrix. A detailed description of the smoothing operation is shown in Figure 7. Figure 7a shows an example of the window selection for smoothing operation, where $w_{t}$ and $w_{a}$ are the window lengths into the time sample and antenna domains, respectively. The selected window matrix is transformed into a column vector of length $w_{t}w_{a}$, as shown in Figure 7b. This process is repeated a total of $n_{a}n_{t}$ times, that is, shifting the window position $n_{a}$ times in the direction of the antenna domain and $n_{t}$ times in the direction of the time sample domain. Then, the smoothing operation for one chirp signal is finished, and it proceeds to all *L* chirp signals, i.e., 1≤l≤L. After the smoothing operation is completed, a 3D data matrix of size $w_{t}w_{a}\times n_{t}n_{a}\times L$, which is denoted by $\tilde{Y}$ is obtained. Then, from the 3D matrix $\tilde{Y}$, the 2D covariance matrix R of size $w_{t}w_{a}\times w_{t}w_{a}$ is calculated as follows [35]:

(10) $$\begin{array}{c} R=\frac{1}{2n_{t}n_{a}}\sum_{l=1}^{L}\left[{\tilde{Y}}_{l}{\tilde{Y}}_{l}^{H}+J\left({\tilde{Y}}_{l}{\tilde{Y}}_{l}^{H}\right)J\right] \end{array}$$

where ${\tilde{Y}}_{l}$ is the *l*th matrix of $\tilde{Y}$, and J is the $w_{t}w_{l}\times w_{t}w_{l}$ exchange matrix. The element of the *i*th row and the *j*th column of J is denoted by J(i,j) and is expressed as:

(11) $$\begin{array}{c} J\left(i,j\right)=\left\{\begin{array}{c} 1,\;\;\mathrm{if}\;\;\;j=n-i+1\\ 0,\;\mathrm{if}\;\;\;j\neq n-i+1 \end{array}.\right. \end{array}$$

The singular value decomposition (SVD) operation is performed on the covariance matrix R. The covariance matrix R is divided into subspaces of the signal and noise, i.e., $U_{\mathrm{signal}}$ and $U_{\mathrm{noise}}$, as follows:

(12) $$\begin{array}{c} R=U_{\mathrm{signal}}DU_{\mathrm{signal}}^{H}+\sigma_{\mathrm{noise}}^{2}U_{\mathrm{noise}}, \end{array}$$

where D is the diagonal matrix of eigenvalues, ${\left(\cdot \right)}^{H}$ is the Hermitian operator, and $\sigma_{\mathrm{noise}}^{2}$ means the noise power. The *M* signal subspaces are $U_{\mathrm{signal}}=\left[u_{1},u_{2},\ldots ,u_{M}\right]$ where $u_{i}$ is the *i*th eigenvector and $U_{\mathrm{noise}}=\left[u_{M+1},u_{M+2},\ldots ,u_{w_{t}w_{l}}\right]$. From $U_{\mathrm{noise}}$, the range-angle pseudo noise spectrum $P_{\mathrm{MUSIC}}\left(R,\theta \right)$ is calculated as follows:

(13) $$\begin{array}{c} P_{\mathrm{MUSIC}}\left(R,\theta \right)=\frac{1}{v{\left(R,\theta \right)}^{H}U_{\mathrm{noise}}U_{\mathrm{noise}}^{H}v\left(R,\theta \right)} \end{array}$$

where v(R,θ) is the steering vector of length $w_{t}w_{a}\times 1$ corresponding to range and DOA. The steering vector v(R,θ) is calculated as v(R,θ)=[v(R)⨂v(θ)] where ⨂ is the Kronecker product operator, v(R) and v(θ) are the range and DOA steering vectors, respectively, and they are expressed as:

(14) $$\begin{array}{c} v\left(R\right)=[1,\exp\left(j4\pi BR/\left(cTf_{s}\right)\right),\exp\left(j8\pi B/\left(cTf_{s}\right)\right),\ldots ,\exp\left(j\left(w_{t}-1\right)4\pi B/\left(cTf_{s}\right)\right)], \end{array}$$

(15) $$\begin{array}{c} v\left(\theta \right)=[1,\exp\left(j\left(2\pi dsin\left(\theta \right)\right)/\lambda \right),\ldots ,\exp\left(j\left(2\pi \left(w_{a}-1\right)dsin\left(\theta \right)\right)/\lambda \right)]. \end{array}$$

**Figure 8:**
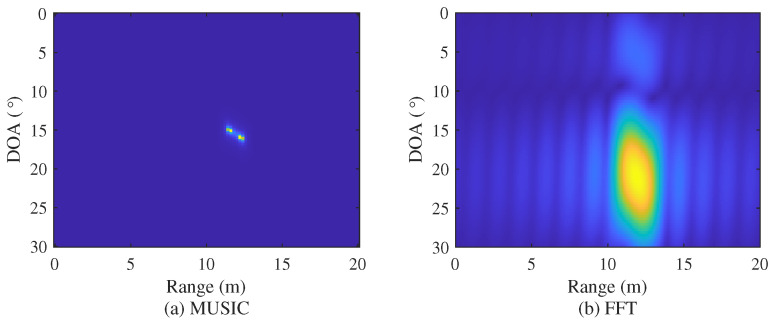
Comparison of resolution between 2D MUSIC and 2D FFT algorithms.

Figure 8 shows the comparison of the resolution between the 2D MUSIC and 2D FFT algorithms. The resolution of the 2D MUSIC algorithm shown in Figure 8a is higher than that of the FFT algorithm shown in Figure 8b. However, the computation complexity of 2D MUSIC algorithm is significantly higher compared with the FFT algorithm. Therefore, in Section 3.3 and Section 4, a low complexity MUSIC algorithm that overcomes the shortcomings of this 2D MUSIC algorithm is introduced.

### 3.3. Low Complexity MUSIC Algorithm Using FFT Estimation

This section introduces a low complexity MUSIC algorithm using FFT estimation [29]. In general, in FMCW radar systems, the sample rate is determined based on the maximum detection range, i.e., the number of sample $N_{s}$ is as follows:

(16) $$\begin{array}{c} N_{s}=\left\lfloor \frac{4B{\hat{d}}_{\max}}{c}\right\rfloor \end{array}$$

where *c* is the speed of the electromagnetic wave.

**Figure 9:**
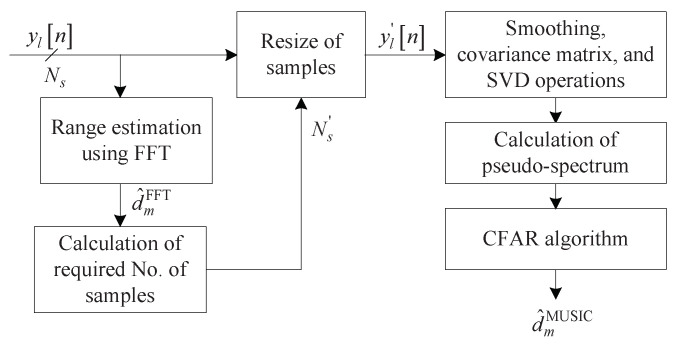
Structure of the low complexity MUSIC algorithm.

However, the target is usually closer than the maximum detection range. Therefore, this algorithm first estimates the approximate range of the target using FFT, selects only the samples necessary for the estimated range, and uses them instead of $N_{s}$ as an input to the MUSIC algorithm. Figure 9 illustrates the structure of the low complexity MUSIC algorithm. The resized number of samples $N_{s}^{\prime }$ is calculated as follows [29]:

(17) $$\begin{array}{c} N_{s}^{\prime }=\left\lfloor \frac{4B{\hat{d}}_{m}^{{}_{\mathrm{FFT}}}}{c}\right\rfloor \end{array}$$

where ${\hat{d}}_{m}^{{}_{\mathrm{FFT}}}$ is the estimated range by FFT. In most cases, since ${\hat{d}}_{m}^{{}_{\mathrm{FFT}}}$ is smaller than $d_{\max}$, the redundant computational complexity is reduced.

However, there is still redundant computational complexity in this algorithm. The case for all regions are considered when calculating the pseudo spectrum. Moreover, the DOA estimation is not considered in this paper. In the next section, in order to overcome this disadvantages of this algorithm, we propose a super resolution algorithm that further reduces the complexity by limiting the region of area in which the pseudo-spectrum is obtained in the area where the targets exist.

## 4. Proposed Subspace-Based Estimation Algorithm for FMCW Radar

In this section, we illustrate the low complexity subspace-based estimation algorithm. The proposed algorithm overcomes the disadvantages of the low complexity algorithm in [29] by limiting the region of the pseudo-spectrum to the area where the targets exist.

**Figure 10:**
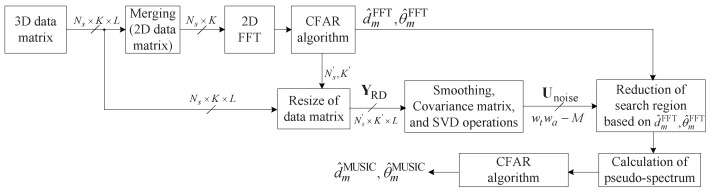
Structure of the proposed algorithm.

Figure 10 shows the structure of the proposed algorithm. The proposed algorithm first estimates range and DOA by 2D FFT. To this end, a 2D data matrix composed of the time sample and antenna domains is generated by merging the 3D data matrix onto the Doppler domain. This 2D data matrix is converted into the range-DOA domain by performing 2D FFT and thus, the estimated range ${\hat{d}}_{m}^{\mathrm{FFT}}$ and the estimated DOA ${\hat{\theta }}_{m}^{\mathrm{FFT}}$ are obtained. These two estimation results ${\hat{d}}_{m}^{\mathrm{FFT}}$ and ${\hat{\theta }}_{m}^{\mathrm{FFT}}$ are used first as a criterion for resizing the data matrix to be input to the MUSIC algorithm and, secondly, employed as a criterion for reducing the search area in the process of the pseudo-spectrum in the MUSIC algorithm.

The resizing criteria for the range, i.e., $N_{s}^{\prime }$ is based on (17). Meanwhile, in the case of $K^{\prime }$, it is determined by the relation between ${\hat{\theta }}_{m}^{\mathrm{FFT}}$ and field of view (FOV) $\theta_{\mathrm{FOV}}$. The FOV according to the distance between adjacent RX antennas $d_{s}$ is expressed as [32]:

(18) $$\begin{array}{c} \theta_{\mathrm{FOV}}=\sin^{-1}\left(\frac{\lambda }{2d}\right). \end{array}$$

From (18), the distance between adjacent RX antennas $d_{s}$ is rewritten as:

(19) $$\begin{array}{c} d_{s}=\frac{\lambda }{2\sin(\theta_{\mathrm{FOV}})}. \end{array}$$

**Figure 11:**
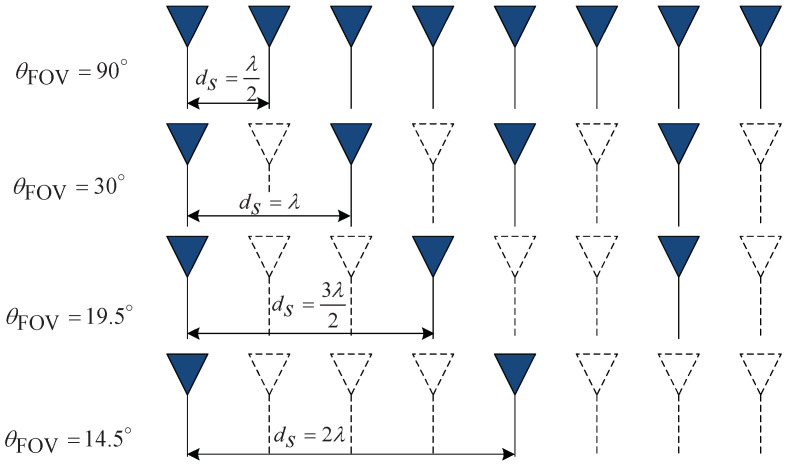
Relation between FOV and the distance between adjacent RX antennas *d* under the condition with the same DOA resolution.

Figure 11 illustrates the relationship between adjacent RX antennas $d_{s}$ under the condition with the same DOA resolution. As the distance $d_{s}$ between the antennas increases, the DOA resolution is maintained even with a small number of antennas, but the FOV is narrowed, and thus, ambiguity occurs. By employing the relation FOV and $d_{s}$ and (20), the $d_{s}$ is obtained as follows: From (18), the distance between adjacent RX antennas $d_{s}$ is rewritten as:

(20) $$\begin{array}{c} d_{s}=\frac{d_{0}\lambda }{2} \end{array}$$

where $d_{0}$ is the integer number indicating the distance between RX antennas, i.e., $d_{0}\in \left[1,2,3,4\right]$. Therefore, $d_{0}$ is calculated by FFT DOA estimation ${\hat{\theta }}_{m}^{\mathrm{FFT}}$ as:

(21) $$\begin{array}{c} d_{0}=\left\lceil \frac{1}{\sin\left({\hat{\theta }}_{m}^{\mathrm{FFT}}\right)}\right\rceil \end{array}$$

where $\left\lceil \cdot \right\rceil$ is the ceil operator. From these results, it is found that there is no degradation of DOA resolution performance, even if a portion of the data matrix is used as in [32]. By doing so, it is expected that the number of data in antenna domain can be reduced.

**Figure 12:**
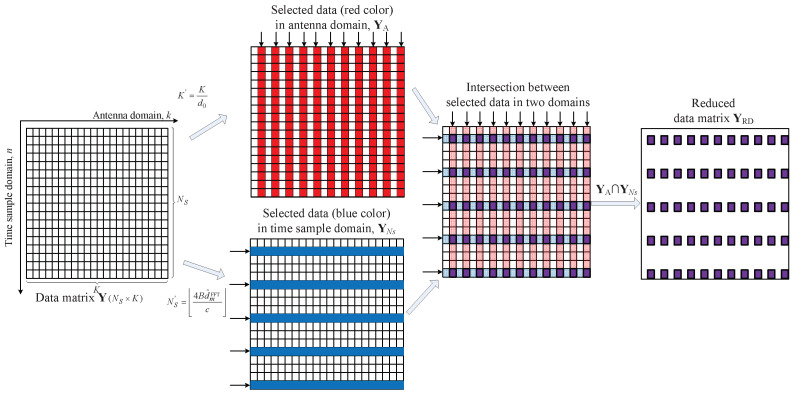
Example of the process of reducing the size of the data matrix of the proposed algorithm.

Figure 12 shows an example of the process of reducing the size of the data matrix to be input into the MUSIC algorithm. The size of data matrix $N_{s}\times K$ becomes $N_{s}^{\prime }\times K{}^{\prime }$, where the reduced number of data in antenna domain is $K{}^{\prime }$. The resizing criteria in the antenna domain of the data matrix are as follows:

(22) $$\begin{array}{c} K^{\prime }=\frac{K}{d_{0}}. \end{array}$$

In Figure 12, $Y_{N_{s}}$ and $Y_{A}$ mean the selected data matrices by (17) and (22), respectively. Finally, the reduced data matrix $Y_{\mathrm{RD}}$ is generated from corresponding to the intersection $Y_{N_{s}}$ and $Y_{A}$. As mentioned above, based on the range-DOA results by 2D FFT estimation, the optimal condition in which the performance degradation of the range-DOA resolution does not occur is obtained.

**Figure 13:**
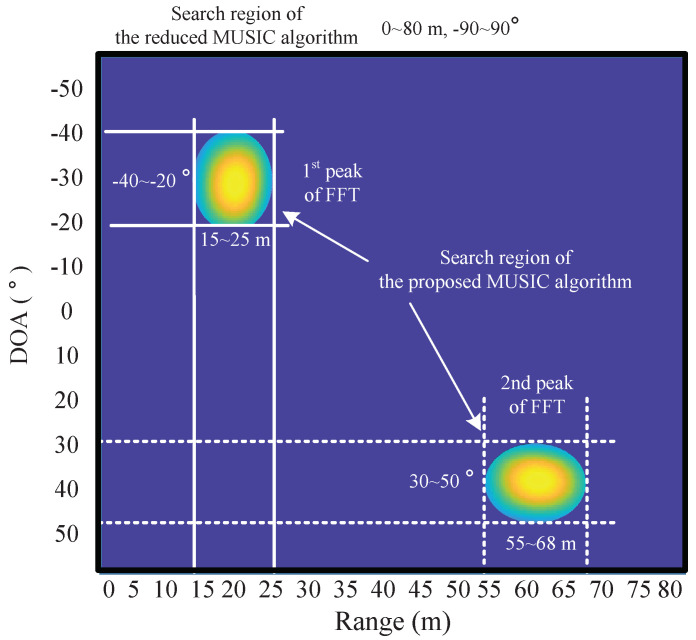
Example of the comparison of the search region between the reduced [29] and proposed algorithms.

Then, the reduced data matrix $Y_{\mathrm{RD}}$ is subjected to smoothing, covariance matrix, and SVD operations, as in the conventional MUSIC algorithm in Figure 6. In these processes, the size of the data matrix is significantly reduced, and thus, the computational complexity required for smoothing, covariance matrix generation, and SVD operation is also significantly reduced compared to the conventional MUSIC algorithm. After that, a process of calculating a pseudo-spectrum based on the noise eigenvector obtained through SVD is performed. In this process, the correlations between the candidate values of range and DOA with the eigenvector of noise are calculated. Since the estimated values are orthogonal to the noise eigenvector, the result of the correlation becomes 0. Therefore, these correlation values appear as peaks because they are located in the denominator. In this process, the proposed algorithm drastically reduces the range of candidate values for calculating the correlation with the noise eigenvector, compared to not only the conventional MUSIC algorithm, but also the reduced MUSIC algorithm [29]. Figure 13 illustrates an example of the comparison of the search region for calculation of the pseudo-spectrum between the reduced MUSIC algorithm [29] and the proposed algorithm. In the conventional and the reduced MUSIC algorithms, all regions are considered as candidate values, as shown in the black line in Figure 13. In other words, a region of $0\leq d\leq d_{\max}$ for range and a region of $-90^{\circ }\leq \theta \leq 90^{\circ }$ for DOA is considered. On the other hand, in the proposed algorithm, the regions of candidate values are limited around the range-DOA values estimated by 2D FFT rather than all regions as shown in the white squares in Figure 13. By doing so, the proposed algorithm significantly reduces the complexity compared to the conventional and the reduced MUSIC algorithms.

## 5. Performance Evaluation

### 5.1. Simulation Results

This section confirms that the complexity of the proposed algorithm is reduced without degrading the performance of the proposed algorithm compared to the conventional and the reduced 2D MUSIC algorithm through the simulation results. The distance between adjacent RX antennas is set to half wavelength, i.e., $d_{s}=\lambda /2$, and the center frequency $f_{c}$ is set to 24 GHz. The complex amplitude ${\dot{a}}_{m,l}$ was independently and randomly generated from uniform distribution and its magnitude, and the phase terms are $0\leq |{\dot{a}}_{m,l}|\leq 1$ and $0\leq ∠{\dot{a}}_{m,l}\leq 2\pi$, respectively. The results of Monte Carlo simulation are averaged over $10^{5}$ estimates. For convenience, the conventional, the low complexity algorithm [29], and the proposed MUSIC algorithms are called ‘conventional algorithm’, ‘reduced algorithm’, and ‘proposed algorithm’, respectively, from now on. The parameter values for the simulation are shown in Table 1.

**Figure 14:**
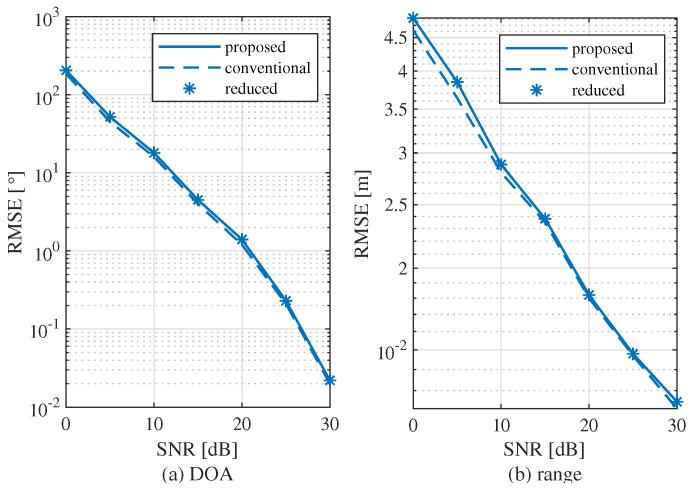
RMSE according to SNR.

Figure 14 shows the root mean square errors (RMSE) of the conventional, the reduced, and the proposed algorithms. Figure 14a,b show the RMSEs of the range and DOA estimations, respectively. From the results, it is shown that the RMSEs of the conventional, the reduced and the proposed MUSIC algorithms are almost the same. This implies that the proposed algorithm has almost no performance degradation compared to the conventional and reduced algorithms, despite the reduced complexity.

### 5.2. Complexity Analysis

In this section, the computational complexity of conventional, reduced, and proposed algorithms is analyzed. In order to analyze the burden of complexity of these algorithms, the required number of multiplications of the main operations is compared [37]. As the main operations to be reflected in the complexity analysis, the generation of the correlation matrix, SVD operation, and pseudo-spectrum operations are employed. For convenience, the variables representing the complexity of the conventional, the reduced, and the proposed algorithms are denoted by $C_{\mathrm{conventional}}$, $C_{\mathrm{reduced}}$, and $C_{\mathrm{proposed}}$, respectively.

The conventional MUSIC algorithm requires that *L* covariance matrices, SVD, noise subspace, and pseudo-spectrum. Hence, $C_{\mathrm{conventional}}$ is calculated as:

(23) $$\begin{array}{c} \begin{array}{ccc} C_{\mathrm{conventional}} & = & \underset{L\;\mathrm{covariance}\;\mathrm{matrices}}{\underbrace{\frac{LKN_{s}\left(KN_{s}+1\right)}{2}}}+\underset{\mathrm{SVD}}{\underbrace{\frac{16}{5}K^{3}N_{s}^{3}}}\\ & + & \underset{\mathrm{Noise}\;\mathrm{subspace}}{\underbrace{\frac{\left(KN_{s}-M\right)\left(KN_{s}+1\right)}{2}}}+\underset{\mathrm{Pseudo}-\mathrm{spectrum}}{\underbrace{KN_{s}\left(KN_{s}+1\right)\left(2N_{\Delta \theta }N_{\Delta R}\right)}} \end{array} \end{array}$$

where $N_{\Delta R}$ and $N_{\Delta \theta }$ are the number of candidate samples of the range region and the DOA region to scan, respectively.

In the case of the reduced MUSIC algorithm, 2D FFT are employed in order to estimate of targets. In addition, the complexity is adaptively changed according to the estimated range and estimated DOA. Therefore, the estimated ranges and the estimated DOAs of the targets are set as average values, assuming that they were uniformly distributed, i.e., it was assumed that $K^{\prime }=K/2$ and $N_{s}^{\prime }=N_{s}/2$. Hence, the $C_{\mathrm{reduced}}$ is calculated as:

(24) $$\begin{array}{ccc} C_{\mathrm{reduced}} & = & \underset{2D\;\mathrm{FFT}}{\underbrace{\frac{N_{R}}{2}\left(K\log_{2}N_{R}+N_{A}\log_{2}N_{A}\right)}}+\underset{L\mathrm{covarianc}\mathrm{ematrices}}{\underbrace{\frac{LKN_{S}\left(KN_{S}+4\right)}{32}}}+\underset{\mathrm{SVD}}{\underbrace{\frac{1}{20}K^{3}N_{S}^{3}}}\\ & + & \underset{\mathrm{Noise}\;\mathrm{subspace}}{\underbrace{\frac{\left(N_{s}-4M\right)\left(N_{s}+4\right)}{32}}}+\underset{\mathrm{Pseudo-spectrum}}{\underbrace{\frac{KN_{s}}{16}(KN_{s}+4)(2N_{\Delta \theta }N_{\Delta R})}} \end{array}$$

In the proposed algorithm, the resizing of data matrix is the same as in the reduced MUSIC algorithm. In order to reflect the reduction of search region for range and DOA regions to scan, let us denote $N_{\Delta R}^{\prime }$ and $N_{\Delta \theta }^{\prime }$ by the reduced $N_{\Delta R}$ and the reduced $N_{\Delta R}$ by the proposed algorithm. The averages of $N_{\Delta R}^{\prime }$ and $N_{\Delta \theta }^{\prime }$ calculated as $E\left[N_{\Delta R}^{\prime }\times N_{\Delta \theta }^{\prime }\right]\ll N_{\Delta R}\times N_{\Delta R}$. Therefore, $C_{\mathrm{proposed}}$ is as follows:

(25) $$\begin{array}{c} \begin{array}{ccc} C_{\mathrm{proposed}} & = & \underset{2D\;\mathrm{FFT}}{\underbrace{\frac{N_{R}}{2}\left(K\log_{2}N_{R}+N_{A}\log_{2}N_{A}\right)}}+\underset{L\;\mathrm{covariance}\;\mathrm{matrices}}{\underbrace{\frac{LKN_{s}\left(KN_{s}+4\right)}{32}}}+\underset{\mathrm{SVD}}{\underbrace{\frac{1}{20}K^{3}N_{s}^{3}}}\\ & + & \underset{\mathrm{Noise}\;\mathrm{subspace}}{\underbrace{\frac{\left(N_{s}-4M\right)\left(N_{s}+4\right)}{32}}}+\underset{\mathrm{Pseudo}\;\mathrm{spectrum}}{\underbrace{\frac{M^{\prime }KN_{s}\left(KN_{s}+4\right)\left(N{}_{\Delta \theta }^{\prime }+N{}_{\Delta R}^{\prime }\right)}{8}}} \end{array} \end{array}$$

where $M^{\prime }$ is the number of peaks of 2D FFT estimation. According to the simulation results, it was confirmed that the average region of the proposed algorithm was only about 0.2% of the case of reduced algorithm when M∈[2,4].

**Figure 15:**
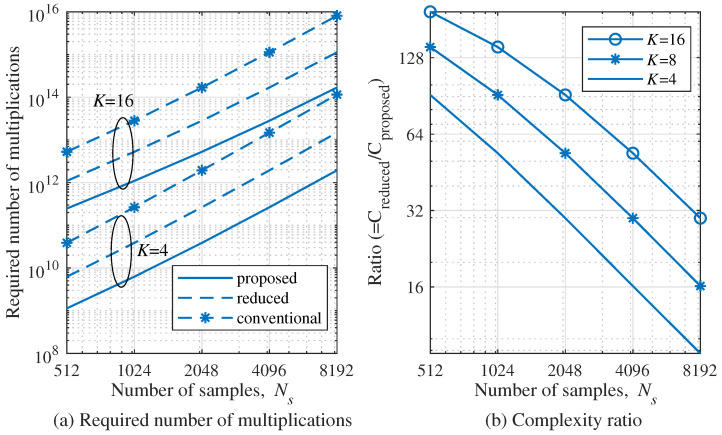
Comparison of the required number of multiplications according to the number of samples $N_{s}$.

Figure 15a shows the required number of multiplications according to the number of samples $N_{s}$ for several numbers of antennas *K*. The number of targets *M* was set to 4, and the number of chirp signals *L* was set to 128. In the case of K=4 and $N_{s}=512$, In the case of $N_{s}=512$ and K=4, the proposed algorithm achieves about 91 and 136 times lower complexity compared to the reduced and the conventional MUSIC algorithms, respectively. As the number of samples $N_{s}$ decreases, the reduction by proposed algorithm also decreases. However, even in the case of $N_{s}=8192$ and K=4, the proposed algorithm achieves about 8.8 and 70 times lower complexity compared to the reduced and the conventional MUSIC algorithms, respectively. In the case of K=16 and $N_{s}=512$, the proposed algorithm achieves about 194 and 217 times lower complexity compared to the reduced and the conventional MUSIC algorithms, respectively. For convenience, Figure 15b shows the ratio of complexities of the proposed and the reduced MUSIC algorithms, i.e., $\mathrm{Ratio}=C_{\mathrm{reduced}}/C_{\mathrm{proposed}}$. From Figure 15b, the complexity of the proposed algorithm is lower than the reduced MUSIC algorithm for $512\leq N_{s}\leq 8,192$ and 4≤K≤16.

### 5.3. Experiments

**Figure 16:**
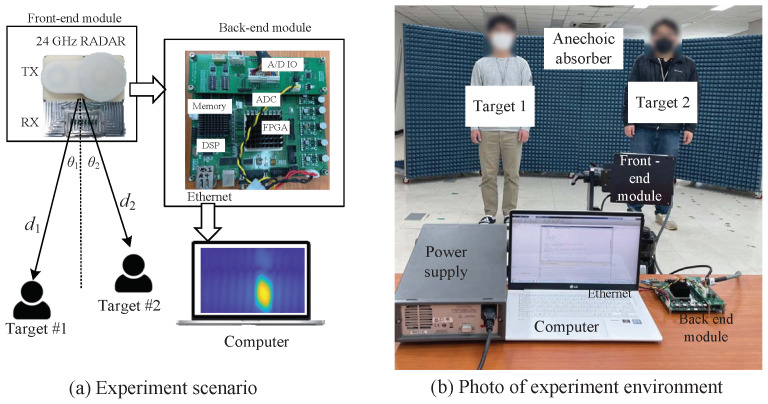
Images of front-end module and back-end module for the experiment.

**Figure 17:**
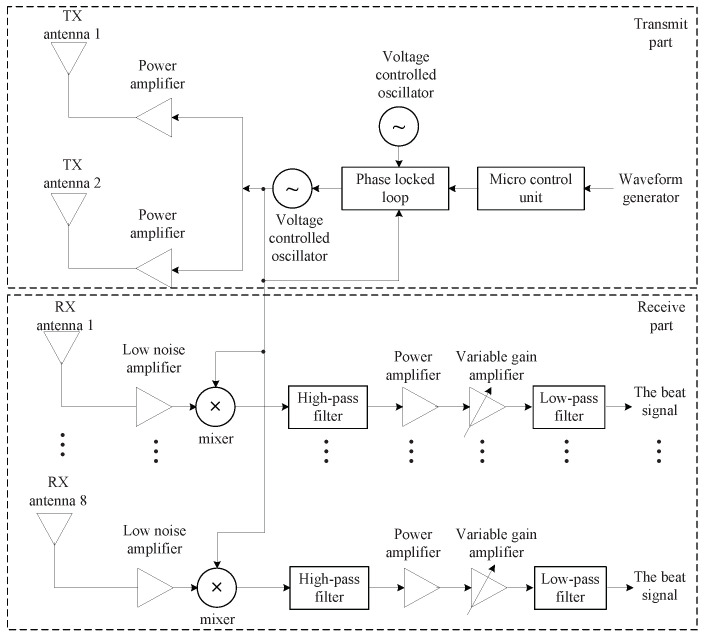
Block diagram of the front end module of 24 GHz FMCW radar for experiment.

In this section, the experimental results are analyzed to check that the proposed algorithm works well under practical conditions. As shown in Figure 16a, the considered radar system consists of the front end module (FEM) part and the back end module (BEM) part, as in [15]. The FEM part consists of the TX and RX parts, as shown in Figure 17. The number of TX antennas is two, and that of RX antennas is eight. The TX part contains the voltage controlled oscillator (VCO), the micro controller unit (MCU), frequency synthesizer, and power amplifiers (PA). The MCU controls the frequency synthesizer with phase-locked loop. The VCO outputs are amplified by PA and then are connected to the two TX antennas. One TX antenna in two TX antennas is selected because they can not work simultaneously. The azimuth angles of two TX antennas cover 26${}^{\circ }$ and 12${}^{\circ }$, respectively. Meanwhile, the RX part includes the low-noise amplifiers, and the high-pass and low-pass filters. The azimuth and elevation of RX antennas cover 99.6${}^{\circ }$ and 9.9${}^{\circ }$. The RX signals are received to the eight RX antennas and The RX signals pass the low noise amplifiers (LNAs) and thus their SNRs are improved. The output of LNAs are multiplied to TX signals and then the outputs of high pass filters are amplified by PA with 6 dB gain and variable gain amplifiers (VGAs) with −2.5 dB to 42.5 dB gain. Then, the outputs pass the low pass filters with 1.7 MHz and finally, the beat signals of the eight channels of FMCW radar are obtained. Meanwhile, the BEM part includes a field programmable gate array and digital signal processing (DSP). The eight beat signals from FEM are converted from analog to digital signals with 20 MHz sampling rate through analog to digital converter. After the external memory is filled, through Ethernet cable, the ADC data is moved to the computer to observe the experiment results. Figure 16b shows the photo of experiment environment. As shown in Figure 16, two persons are employed as targets. The ranges and angles of the two targets were set close enough to be indistinguishable by the FFT estimator.

**Figure 18:**
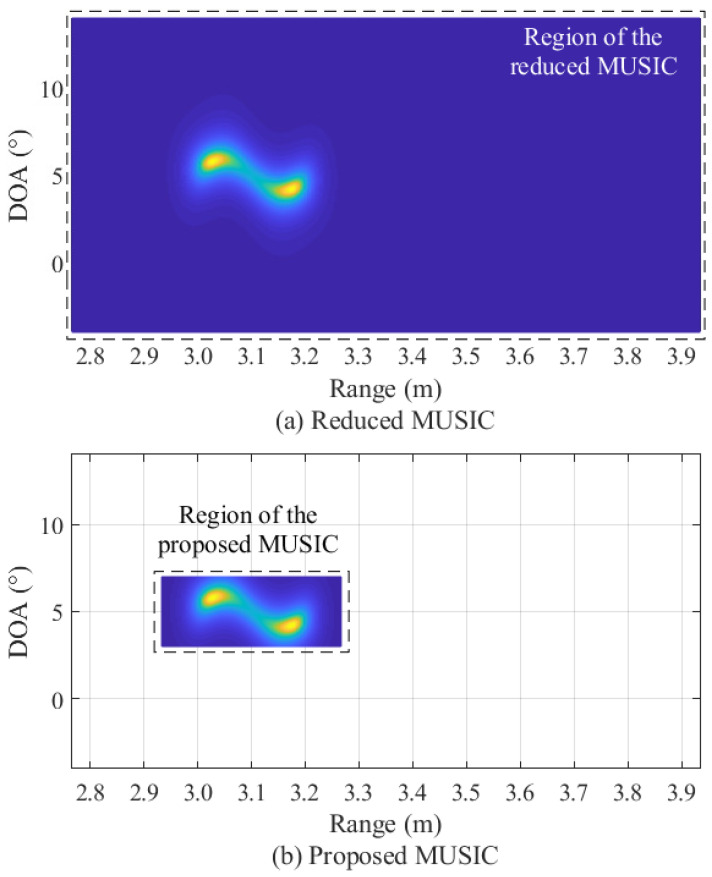
Experimental results of the reduced and proposed MUSIC algorithms (3.03 m and 3.17 m).

The experiment results of the reduced algorithm and the proposed algorithm are shown in Figure 18. Since the reduced algorithm considers all regions in the process of obtaining a pseudo-spectrum, the range-DOA map covers the entire region, as shown in Figure 18a. On the other hand, in Figure 18b, in the results of the proposed algorithm, only the region where the target exists was considered. From these results, it is confirmed that the range-DOA estimation results of the two algorithms are the same. This implies that the proposed algorithm reduces the complexity, while achieving a similar performance to the conventional algorithm.

## 6. Conclusions

This paper proposed a low-complexity 2D MUSIC algorithm by reducing the region of the pseudo-spectrum and the input of the MUSIC algorithm. It was shown that the computational complexity can be reduced by limiting the search area to scan based on the FFT estimator, which is a representative low-complexity algorithm. The simulation results showed that the complexity reduction of more than 100 times was achieved by the proposed algorithm. The effectiveness of the proposed algorithm was verified by simulations and experiments using 24 GHz FMCW radar systems. Therefore, the proposed algorithm is one of solutions to solve the high complexity of the subspace-based algorithms.

## 7. Discussion

This section covers the limitations and further work on the proposed algorithm. In the proposed algorithm, when the number of peaks of 2D FFT is very large, the search regions to be partially calculated also increase. As a future study, we plan to analyze how many peaks achieve lower complexity compared to previous algorithms. Furthermore, the implementation and pipeline structure of the proposed algorithm, as an FPGA will be dealt with later.

## Figures and Tables

**Table 1:** Parameter values for simulations.

Parameter	Value
Center frequency, $f_{0}$	24 GHz
Bandwidth, *B*	100 MHz
Chirp duration, *T*	100 μs
SNR	10 dB
Number of samples, $N_{s}$	66
Sampling frequency, $f_{s}$	0.67 MHz

## Data Availability

Not applicable.
